# Current and future trends in Russian Rheumatology Care and Research

**DOI:** 10.31138/mjr.28.4.201

**Published:** 2017-12-22

**Authors:** Elena V. Tchetina

**Affiliations:** Immunology and Molecular Biology Laboratory, Nasonova Research Institute of Rheumatology, Moscow, Russia

**Keywords:** Rheumatic diseases, research, development, Russia

## Abstract

This short article provides a description of the present state of rheumatology care and research in Russia and discusses opportunities for development and co-operation.

Rheumatic diseases (RDs) in Russia represent the second most common pathology after cardiovascular disorders. Although RDs include more than 200 diseases and syndromes, the most common in Russia are osteoarthritis (OA), rheumatoid arthritis (RA), gout, and low back pain, largely similar to those seen in most Western societies. Interestingly, the prevalence of RA appears to have increased from 0.42% to 1% in the Russian population since the last large-scale epidemiologic study, which was conducted 25 years ago in the former USSR; this is an opposite trend to that seen in most Western countries^1^ and is similar to other reports that have indicated that RA prevalence has risen in Europe and North America due to the underlying aging population^2^ and increasing patient survival.^3^ This would be interesting to study in comparative collaborative research. Approximately 10% of the Russian population is presently affected by RDs.^4^ The major factors responsible for the increased prevalence of RDs in Russia include increased mutation activity associated with environmental pollution and an augmented incidence of concomitant disorders such as obesity, diabetes mellitus, hypertension, and others.

Recent large epidemiologic study including 76,000 subjects from 18 regions of the Russian Federation revealed that statistical indices are significantly lower (up to 5-fold) than real prevalence of RDs among adults registered according to the attendance in clinics.^5^ Besides, individual federal regions demonstrate different dynamics. For example, in Central Russia, the incidence of almost all RDs excluding osteoporosis decreased, while in the Western region, an increase in the incidence of RA and OA during one year of follow-up was observed.^6^

Russia is a large territory and has a multinational population. This provides the opportunity to study different disease subtypes and phenotypes associated with genetic variability, national psychological traits, various climates, ecological conditions, and environmental factors. However, all these possibilities are limited by the insufficient funding of rheumatic research. Therefore, such type of research is a subject for future studies.

Recently, Russia is developing a three-level standardized system for management of RD patients involving primary medical care, specialized treatment protocols, and rehabilitation technologies developed by the leading rheumatologic centers.

Russia has great tradition in rehabilitation. Recent studies on combination of “treat to target” therapies with concomitant rehabilitation program have demonstrated that this approach is more efficient than pharmacological therapy alone primarily for early RA patients. It involves local air cryotherapy, special gymnastics for joints, ERGO therapy and various types of orthoses. Regular high-intensity dynamic training has been shown to reduce pain and decrease the amount of prescribed DMARDs.^7^

In the international scene, the approach to RA management has changed significantly in the last 2 decades. Important improvements in the assessment of RA in clinical practice have taken place (e.g., the use of standardized assessments of disease activity and severity such as the DAS28 and the utilization of novel imaging modalities such as ultrasound and MRI). Early access to rheumatology services and early disease recognition have enabled early, aggressive management strategies utilizing the “window of opportunity” for effective suppression of inflammation.^8^ The therapeutic pyramid was inverted, from a “go low, go slow” to earlier and more aggressive disease management.^9^ Combinations of conventional synthetic DMARDs with corticosteroids^10^, and an increase in the doses of traditional drugs, such as methotrexate,^11^ enabled this. The addition of the anti-TNF and later other biologic DMARDs, targeting specific cytokines (e.g., IL-6 and IL-1), of anti-B cell therapies (e.g., rituximab) and therapies targeting other important pathways of inflammation (e.g., abatacept) in our therapeutic armamentarium has been a true revolution which has enabled a significant proportion of our RA patients to achieve remission or at least a low disease activity state – particularly when used in a “treat-to-target” (T2T) fashion. These developments are now being followed with the introduction of small-molecule anti-inflammatory compounds such as inhibitors of Jak-family kinases, Syk tyrosine kinase, and p38 MAP kinase^12,13^ that target intracellular signal transduction pathways. In contrast to biologics, the small molecules are orally administered; they have a short *in vivo* half-life and are stable during storage. Furthermore, small molecules targeting non-kinase targets such as calcineurin, mTOR, adenosine receptors or ion channels are currently under investigation and may provide more therapeutic avenues in the future.

At present, eight drugs related to biological therapy of RA are registered and used for patient treatment in Russia.^12^ The Russian Association of Rheumatologists has priority research programs such as RADICAL, REMARKA, and ETALON for studies of methotrexate and biological agents for RA treatment.^14^ For example, the RADICAL program is aimed to assess the disease activity, functional status, and X-ray changes in patients with early RA treated by traditional DMARDs and biological agents. It was demonstrated that although low disease activity after MTX treatment positively affects functional status of a patient, irreversible structural joint changes continue to develop.^15^ ETALON study investigates changes of the quality of life during treatment of RA patients with etanercept.^16^

When the general strategy of “treat-to-target” therapy was designed, the REMARCA study - aiming to determine the efficacy of combined MTX and biological therapy treatment for RA patients to achieve clinical remission or low disease activity - was launched in Russia. The specificity of the Russian T2T study was the predominant use of subcutaneous MTX in high concentration and its fast dose increase, the absence of glucocorticoid, early involvement of biological therapy, and stringent remission criteria.^17^ Subcutaneous MTX delivery demonstrated higher efficacy and better tolerance compared to pills.^18^ Since then, 76% of patients included in REMARCA study reside in Central Russia.^19^ It will further be extended to other Russian territories.

However, only large rheumatological centers in Russia have good opportunities to treat RA patients involving expensive biological drugs supported by the governmental program for high-technology medical care.^14^ Furthermore, there are some problems in the early identification of the disease in the Russian Federation. This is caused by the low level of medical aid appealability, due to the remoteness of huge territories from medical centers and limited communication capacities between them. In addition, funding limitations, lack of family doctors, and an insufficient number of rheumatologists in the primary outpatient care service results in misdiagnosis, which produces incorrect initial treatment. These delays may lead to a loss of the “window of opportunity” for many patients with RDs.^5^

The access to therapies could be simplified when less expensive Russian analogues of known antirheumatic drugs are developed and distributed in Russia. Presently, Acellbia, the first Russian bioanalogue of the drug Mab-Thera (Rituximab) has demonstrated therapeutic equivalence in a BIORA study.^20^ In addition, original biological agents for RD treatment were developed by the Russian pharmaceutical company BIOCAD. These are BCD-085, a humanized monoclonal antibody to IL-17 (Phase II), BCD-089, a humanized monoclonal antibody to IL-6 receptor (Phase I), and BCD-121, a humanized monoclonal antibody to both TNFα and IL-17.^21^

Furthermore, registries of Russian patients with different RDs and the updated national recommendations for treatment of individual RDs are now available. For example, the first registry (ARBITR) was launched in Russia in 2005 when the first agent for biological therapy, an anti-TNFα drug (infliximab) was registered.^14^ Starting in 2011, it was transformed into the OREL registry, which quickly developed as an internet-based project and now includes 3,276 RA patients.^17^

The OREL registry contains the most comprehensive data about RA patients in Russia.^14^ The registry demonstrates a significant prevalence of RA in city residents over rural, although this may also be associated with the concentration of rheumatologic care centers in big cities, and their limited availability in the villages. Moreover, the OREL registry includes fewer smokers compared to other registries from European countries and the United States, and contains the most detailed information on comorbidities,^14^ which prevent improvements in the quality of life during RA treatment by DMARDs.^22^ The registry helps to identify groups of patients with variable disease courses and develop specific treatment protocols for each geographical region. This registry permits regulation, prevents an overuse of governmental financial support, and stays in limits with regional economic possibilities.

To provide a high professional level of rheumatologists, Russia has introduced a system of continuous additional professional education. This system involves collaboration among heads of regional rheumatology departments with federal research and educational unities, which facilitates changes in programs for specialist teaching with regional specificity. Additionally, a special program for the education of nurses with backgrounds in rheumatology has been developed.^14^

Another approach to improve RA treatment is an original method of Structured Programs for RA patients, involving training for patients to self-evaluate their disease activity by counting the number of tender and swollen joints between visits to the doctor.^23^

Starting in 2000, Russia has conducted multicentral placebo-controlled clinical studies of new antirheumatic drugs in collaboration with leading pharmaceutical companies. Aiming to standardize of the evaluation approaches for the international efficacy and safety of biological therapies, Russian rheumatologists were involved in the CERERRA project for an anti-B-cell agent (Rituximab) investigation in 2011.^24^

As Russian rheumatologic research is focused on the implication of the achievements of fundamental biomedical studies in a clinical setting, collaborative research with other countries is welcomed in relation to genetic and immunological heterogeneity studies of RDs and the identification of risk factors including genetic predisposition, epigenetic disturbances, and environmental factors. Another point of collaboration could include the development of new biological therapies using new targets such as interleukins 23, 21, 22, and 20, as well as the development of the biological agents inactivating two proinflammatory cytokines simultaneously.^21^

In the last decade, the mechanisms of RA regulation at a molecular level were intensively explored using gene expression studies.^25,26^ These studies have also started recently in Russia. For example, it was revealed that high baseline autophagy-related ULK1 gene expression in peripheral blood indicates a good response in respect to pain in patients treated with rituximab.^27^ The higher radiographic joint destruction associated with rheumatoid factor positivity is accompanied by the upregulation of MMP-9 and cathepsin K gene expression in the PBMCs of RA patients treated with methotrexate.^28^ Furthermore, the expressions of MMP-9 and ULK1 indicate disease activity, while increased baseline gene expressions of RUNX2, p21 and caspase 3 in the peripheral blood might predict better responses to MTX therapy.^29^ These studies will help to identify signalling pathways driving the disease process, and new therapeutic targets to address the primary cause of RA, facilitating prediction of disease onset, course, and outcome.

In the group of musculoskeletal disorders, osteoarthritis (OA) is considered the most prevalent and is a leading cause of disability and pain.^30^ Presently, approximately 10% of the world’s population (60 years or older) have clinical manifestations similar to OA.^31^ As OA incidence and prevalence increase with age, longer life expectancy might cause an increase in the number of OA patients in the future.^32^ In Russia, OA is also the most common joint disease, with 4 million affected individuals.

As early diagnosis and appropriate management can minimize the effect of OA,^33^ knowledge of the intrinsic mechanisms of the disease development and progression is of particular importance.

In view of this, we recently presented evidence for the involvement of developmental mechanisms in articular cartilage degradation in OA using human specimens.^34,35^ We observed profound cellular phenotypic changes associated with alterations in gene expression in articular chondrocytes prior to overt cartilage matrix degradation, which can be monitored histologically. This result suggests that articular chondrocyte phenotype modification can be recognized very early in the disease at the gene expression level.

The classical definition of osteoarthritis as a wear-and-tear, non-inflammatory disease has recently transitioned to an inflammatory disorder on a spectrum between normal and RA. It has been shown that the OA disease pathogenesis is driven by an early innate immune response that progressively catalyzes degenerative changes that ultimately lead to an altered joint microenvironment. Although mechanical factors might have a role in traumatic, established or late OA, systemic factors have a substantial effect on the joint structure in preclinical OA. Systemic processes such as inflammation, aberrant metabolic regulation and obesity control OA pathogenesis. It was also recently suggested that early OA strongly requires routine diagnostic investigations designed to detect the machineries of metabolic syndrome.^36^

Metabolic syndrome is defined as the association of components that independently increase the risk of cardiovascular events including abdominal adiposity, diabetes mellitus or insulin resistance, high cholesterol and high blood pressure, and could be an independent risk factor for OA. It appeared that 59% of OA patients possess metabolic syndrome compared to 23% of the general population.^37^ Moreover, obese patients with metabolic syndrome have increased risk of incidence and severity of knee and hand OA and increased OA pain.^38^ The key mechanisms of metabolic stress development observed in OA include elevation of adipokines and free fatty acids in joint tissues, oxidative stress, hyperglycaemia, and thrombosis of subchondral bone vasculature.^39^ Therefore, dietary therapy combined with physical exercise and bariatric surgery is an effective treatment for both obesity and the associated metabolic disturbances such as knee OA. At the molecular level, cellular energy and nutrient sensors such as AMP-activated protein kinase (AMPK), sirtuins (SIRTs), and the mechanistic target of rapamycin (mTOR) determine cellular response to amounts of nutrients and changes in cellular energy balance.^37,40^ The development of metabolic syndrome is associated with chronic excess of nutrients. Concomitant dysregulation of these sensors in the articular cartilage involves decreased function of AMPK and SIRT and an increase in mTOR expression and activity.^37^ Thus, activation of AMPK and SIRTs and inhibition of mTOR signalling by dietary restriction might be favourable.^41^

The studies on the association of OA articular cartilage degradation and changes in chondrocyte metabolism have demonstrated that suppression of the excessive collagenase-mediated type II collagen cleavage in OA cartilage by deferoxamine, an iron chelator with anabolic potential, inhibits proinflammatory cytokine and metalloproteinase expression, and reverse phenotypic changes. The concomitant upregulation of type II collagen (CO-L2A1) and pro-anabolic TCA-related gene expression points to a potential for the availability of energy generating substrates required for matrix repair by end-stage OA chondrocytes. However, this might be prevented by high whole-body energy requirements indicated by elevated AMPK expression in the peripheral blood mononuclear cells (PBMCs) of OA patients.^42^

Therefore, the expression of genes associated with global cell survival and function measured in whole blood of OA patients might indicate disease activity. With this regard, it was shown that increased gene expression of mTOR in PBMCs isolated from OA patients was related to the presence of synovitis and was seen in all patients requiring joint replacement. The patients with low expression of mTOR experienced more pain during joint function and had increased joint stiffness.^43^ The observed differences in the disease activity of OA patients based on gene expression analysis of peripheral blood and evidence for the involvement of metabolic syndrome in disease pathogenesis indicate that osteoarthritis is not only a disease of the joints, but involves the whole body. These issues should be considered for appropriate treatment.

Overall, physicians in Russia have similar challenges with other European practitioners. These challenges are to diagnose the disease appropriately and to promptly inhibit the inflammatory process and joint degradation. In view of this, we can learn from Europe how to organize a standardized healthcare system with a network of family doctors. We are presently creating this system in Russia.
